# Effect of Pravastatin on Placental Expression of Epidermal Growth Factor-like Domain 7 in Early-Onset Pre-Eclampsia: A New Potential Mechanism of Action

**DOI:** 10.3390/biomedicines12081929

**Published:** 2024-08-22

**Authors:** Silvia Salvi, Stefano Fruci, Valentina Lacconi, Federica Totaro Aprile, Roberta Rullo, Heidi Stuhlmann, Antonio Lanzone, Luisa Campagnolo, Micol Massimiani

**Affiliations:** 1 UOC di Ostetricia e Patologia Ostetrica, Dipartimento di Scienze della Salute della Donna, del Bambino e di Sanità Pubblica, Fondazione Policlinico Universitario A. Gemelli, IRCCS, L.go Agostino Gemelli 8, 00168 Rome, Italy; silvia.salvi@policlinicogemelli.it (S.S.); stefano.fruci@policlinicogemelli.it (S.F.); roberta.rullo@guest.policlinicogemelli.it (R.R.); antonio.lanzone@unicatt.it (A.L.); 2 Dipartimento Universitario di Scienze della Vita e Sanità Pubblica, Sezione di Ginecologia ed Ostetricia, Università Cattolica del Sacro Cuore, Largo Francesco Vito 1, 00168 Rome, Italy; federica.totaroaprile01@icatt.it; 3 Departmental Faculty of Medicine, Saint Camillus International University of Health Sciences, Via di Sant’Alessandro 8, 00131 Rome, Italy; valentina.lacconi@uniroma2.it; 4 Department of Biomedicine and Prevention, University of Rome Tor Vergata, Via Montpellier 1, 00133 Rome, Italy; 5 Department of Cell and Developmental Biology, Weill Cornell Medical College, 1300 York Avenue, Box 60, New York, NY 10065, USA; hes2011@med.cornell.edu

**Keywords:** pre-eclampsia, pravastatin, EGFL7, notch signaling, chorionic villi, placenta

## Abstract

The primary intervention for pre-eclampsia (PE) remains iatrogenic delivery, which can be very preterm and not optimal for the fetus. Although many efforts have been made to prevent and manage PE, there is still a dearth of drugs to treat its pathophysiological progression. Pravastatin (PRA), a hydrophilic statin, has gained interest for the prevention and treatment of PE. The aim of the present study was to evaluate the ability of PRA to modulate factors involved in placentation, such as Epidermal Growth Factor-Like Domain 7 (EGFL7), in human chorionic villous culture from healthy controls and women with PE. A total of 18 women were enrolled: 10 controls and 8 cases. Chorionic villous explants were maintained in culture for 24 h with or without 10 μM Pravastatin, and the expression of EGFL7 and NOTCH1 pathway members was evaluated by qRT-PCR and Western blot analysis. The rationale of the present study was to establish an ex vivo model to identify potential different responses to PRA treatment of chorionic villous explants in order to clarify the molecular mechanism of PRA in the prevention and treatment of PE and to predict whether there are specific clinical conditions that modulate the response to the drug treatment. Within PE patients, two different groups were identified: the high responders, whose villous cultures exhibit significantly increased expressions of the EGFL7 and Notch pathways after PRA incubation; and the low responders, who are high-risk PE patients in which prophylaxis failed to prevent PE and PRA was not able to modulate EGFL7 expression. In conclusion, we identified EGFL7 as a new factor regulated by PRA, placing interest in early discrimination between low- and high- risk women, in which the well-known pharmacological prophylaxis seems to be ineffective, and to explore new potential prevention strategies.

## 1. Introduction

Maternal endothelial dysfunction is the main pathophysiological mechanism responsible for systemic complications and the clinical scenario of pre-eclampsia (PE) [1,2]. An imbalance of circulating levels of proangiogenic (Placental Growth Factor: PlGF; Vascular Endothelial Growth Factor: VEGF) and antiangiogenic factors (soluble Fms-like tyrosine Kinase 1: sFlt-1) has been detected in PE [3]; thus, their assessment (sFlt-1:PlGF ratio) appears clinically relevant in disease onset prediction, severity, and timing of delivery [4,5,6,7].

Two decades ago, we and others identified a new secreted angiogenic factor, Epidermal Growth Factor-Like Domain 7 (EGFL7), whose expression was initially believed to be exclusively restricted to endothelial cells [8,9,10]. However, we later demonstrated that EGFL7 is expressed in the human placenta, not only in endothelial cells of the chorionic villi, but also in the syncytiotrophoblast and cytotrophoblast cells. We also highlighted the ability of this secreted factor to regulate the migration and invasion of the trophoblast through the activation of mitogen-activated protein kinase (MAPK), phosphatidylinositol 3-kinase (PI3K), and NOTCH signaling pathways [11,12]. Moreover, we previously provided evidence that circulating levels of EGFL7 are significantly higher in patients affected by PE when compared to normal pregnancies, while EGFL7 placental levels in pre-eclamptic women are lower than those measured in healthy control placentas [11,13]. Consistent with these results, villous explant cultures obtained from placentas affected by PE displayed increased release of EGFL7 in the culture medium when compared to those from normal placentas. These results suggest that the increased release of placental-derived EGFL7 and its increased circulating levels, with the possible contribution of diffuse damaged maternal endothelial cells, are associated with the clinical manifestation of PE [13].

To this day, elective therapy for PE requires early delivery with removal of the placenta. Although early delivery is always beneficial for the mother, it may not be optimal for the fetus. Among the pharmacological approaches, one of the most discussed ones is certainly represented by the use of statins. Pravastatin (PRA) is a lipid-lowering drug with hydrophilic activity widely employed to reduce the risk of cardiovascular events in the general population [14]. Indeed, PE shares many pathophysiological characteristics with cardiovascular diseases, such as inflammation and endothelial dysfunction. Based on these premises, the use of statins has gained interest for the prevention of PE. Statins are competitive inhibitors of hydroxymethylglutaryl coenzyme A (HMG-CoA) reductase, the rate-limiting enzyme in cholesterol biosynthesis. Indeed, statins decrease intrahepatic cholesterol levels, leading to increased expression of low-density lipoprotein (LDL) receptors and the reuptake of circulating lipids. Epidemiological data collected to date suggest that statins have no major teratogenic effects in pregnancy and, among these, those with the best pharmacokinetic and safety profile are hydrophilic statins, such as PRA [15,16,17,18,19,20,21,22]. In the last years, in vitro studies, case reports and some clinical trials have highlighted the potential of PRA in the prevention and treatment of PE [15,16,17,18,19,20,21]. Despite the presence of conflicting data in the literature on this matter, it is believed that PRA can play an important role in the prevention of PE when administered early in high-risk patients [20,22].

The aim of our pilot observational study was to assess the ability of PRA to modulate EGFL7 expression in human chorionic villous explant culture from both uncomplicated pregnancies and from women with PE, in order to evaluate pleiotropic effects of this molecule that can be used in the prevention and treatment of PE.

## 2. Materials and Methods

### 2.1. Study Population, Setting and Data Collection

This is a pilot observational study conducted at Fondazione Policlinico Universitario Agostino Gemelli, IRCCS and the Department of Biomedicine and Prevention, University of Rome Tor Vergata, Italy, between May 2019 and June 2023. A total of 18 women were enrolled: 10 controls and 8 cases. Healthy pregnant women with uncomplicated pregnancies and normally grown fetuses were included as controls. Cases (PE) included only women with early-onset pre-eclampsia, diagnosed before 34 gestational weeks and associated with fetal growth restriction (FGR), defined according to the International Society for the Study of Hypertension in Pregnancy (ISSHP) 2018 Guidelines [23]. FGR was defined as a fetus with an estimated weight or abdominal circumference < 3rd percentile for gestational age or with an estimated weight or abdominal circumference < 10th percentile and with abnormal umbilical artery pulsatility index (UA PI) > 95th percentile, according to the definitions reported by Gordjin et al. [24].

The inclusion criteria for both groups were singleton pregnancies with spontaneous conception; certain gestational age; absence of fetal malformations and/or chromosomal abnormalities and/or congenital fetal infections; maternal age > 18 years; and ability to express informed consent. The exclusion criteria were as follows: multiple gestations; medically assisted procreation procedures of the heterologous type; maternal comorbidities influencing the diagnosis and/or evolution of PE (nephropathy, heart disease, antiphospholipid antibodies syndrome); age < 18 years; language barrier; and inability to express informed consent.

According to the hospital protocol, women with PE received anti-hypertensive treatment as needed, steroid prophylaxis for fetal prematurity and magnesium sulfate administration immediately before delivery in case of gestational age < 32 weeks and/or in case of PE with severe features. PE prophylaxis with low-dose aspirin (LDA) and/or low-molecular-weight heparin (LMWH) was performed, when indicated, according to maternal obstetric or pathological past history.

Maternal data, including maternal age, BMI (body mass index), gravidity and parity, mode of conception, past medical and obstetric history, pharmacological treatment, and Doppler findings at diagnosis and delivery, were collected. The collected perinatal variables analyzed were birthweight (g); birthweight centile; neonatal gender; Apgar score at first and at fifth minute; and admission to neonatal intensive care unit (NICU). Birthweight percentile was calculated according to Youdkin et al. [25]. Neonates whose birthweight was lower than 10th percentile were defined as being small for gestational age (SGA).

All placental tissues of women with PE were sent for pathologic examination and analyzed according to the Amsterdam Placental Workshop Group Consensus Statement [26].

### 2.2. Ex Vivo Chorionic Villous Explant Cultures

For both groups, collection of the placenta was performed after caesarean delivery. Explants of chorionic villi were prepared as described by Miller et al. [13,27]. Briefly, 1 cm^3^ villous sections were isolated postdelivery from the parasagittal plane with respect to the umbilical cord insertion of each placenta. Sections were extensively washed in phosphate-buffered saline (PBS, Corning, Mediatech, Manassas, VA, USA) to remove maternal blood and either immediately frozen in liquid nitrogen or cultured for 24 h in 1 mL of RPMI 1640 medium (Corning), supplemented with 2 mM L-glutamine (Lonza, Milan, Italy), 50 U/mL penicillin and 50 mg/mL streptomycin (Lonza), and 1 mM sodium pyruvate (Sigma-Aldrich, Saint Louis, MO, USA), with or without 10 μM PRA (Sigma-Aldrich), in 24-well plates at 37 °C and 5% CO_2_. For each patient, 30–40 mg of villi per well was cultured in 24-well plates (three wells for each condition, − and + PRA). PRA concentration was chosen based on preliminary dose-response experiments for the identification of the lowest effective concentration, according to the literature data [28,29,30,31]. Phase-contrast images were taken under a Leitz Diavert microscope connected to a Nikon DS-Fi1 camera. After 24 h of culture, chorionic villous explant cultures were collected, frozen in liquid nitrogen, and stored at −80 °C until analysis.

### 2.3. qRT-PCR Analysis

RNA from chorionic villous explant cultures was extracted using the TRIZOL Reagent (Roche Diagnostics GmbH, Mannheim, Germany), according to the manufacturer’s protocol. RNA quality was examined by evaluating the presence of ribosomal RNA bands in agarose gels. RNA was reverse-transcribed using random primers and the QuantiTect Reverse Transcription Kit (Qiagen, Hilden, Germany), following the manufacturer’s specifications. Gene expression was measured using iTaq Universal SYBR Green Supermix (Biorad Laboratories, Hercules, CA, USA). qRT-PCR was performed using the LightCycler 96 Real Time PCR System (Roche Diagnostics GmbH). Differences in gene expression were quantified using the ΔΔCt method with normalization to *18S* ribosomal RNA gene. Specific primers for *EGFL7*, *NOTCH1*, hairy and enhancer of split-related protein 1 (*HEY1*), hairy and enhancer of split-related protein 2 (*HEY2*), and 18S were designed using Primer Express software (Applied Biosystems in Life Technologies, Monza, Italy), and their efficiency was tested using standard curves. Primer sequences, amplicon size, and gene accession number are listed in Table 1.

### 2.4. Western Blot Analysis

Flash-frozen chorionic villous explant cultures were homogenized in lysis buffer (50 mM Tris–HCl pH 7.5, 150 mM NaCl, 0.5% NP-40, 5 mM ethylenediaminetetraacetic acid (EDTA), 0.5% sodium deoxycholate, 1 mM phenylmethylsulfonyl fluoride, 20 mM β-glycerophosphate, 1 mM sodium orthovanadate) containing an EDTA-free protease inhibitor cocktail (Roche, Penzberg, Germany). The Bradford assay was used to determine protein content. Protein samples (40 μg) were separated by electrophoresis on 10% (*v*/*v*) sodium dodecyl sulfate polyacrylamide gel electrophoresis (SDS–PAGE) gels and transferred to polyvinylidene difluoride (PVDF) Transfer Membrane Hybond^TM^ (Amersham Biosciences). Nonspecific antibody binding was prevented by incubating the membrane in 5% (*w*/*v*) non-fat dry milk in Tris-buffered saline (TBS) containing 0.1% (*v*/*v*) Tween 20 (TBS/T) for 1 h at room temperature. Membranes were then incubated overnight at 4 °C with rabbit anti-EGFL7 (Abcam, Cambridge, UK, cat. ab256451, 1:1000) or mouse anti-GAPDH (clone 6C5, Santa Cruz, CA, USA, cat. sc-32233, 1:2000), all diluted in TBS/T with 5% (*w*/*v*) bovine serum albumin (BSA). Horseradish peroxidase conjugated secondary anti-rabbit and anti-mouse antibodies (Amersham Biosciences) were diluted in 5% (*w*/*v*) non-fat dry milk containing TBS/T (1:10,000 and 1:5000, respectively) and were incubated with the membranes for 1 h at room temperature. Immunoreactive bands were detected by LiteAblot Turbo chemiluminescent substrate (Euroclone, Pero, Italy) according to the manufacturer’s protocol. Densitometric analysis of the bands was performed using ImageJ ij154-win-java8 software.

### 2.5. Statistical Analysis

Continuous variables were expressed as mean ± standard error of the mean (SEM), or as median with interquartile range (IQR) if not normally distributed, and categorical variables were displayed as frequencies. Data were analyzed by using either parametric (Student’s *t* test) or nonparametric (Mann–Whitney and Kruskal–Wallis tests) tests, according to the results obtained after normality test (the Shapiro–Wilk test) and variance analysis (using the Brown–Forsythe or F test, as appropriate). Dunnett’s test was used after Kruskal–Wallis test. The software used included GraphPad Prism 8, SigmaPlot 12.0, and the Statistical Package for Social Science (SPSS) Version 25. *p*-values < 0.05 were considered significant.

## 3. Results

### 3.1. Maternal and Neonatal Clinical Data

We included in our analysis 18 patients, represented by 10 healthy controls and 8 women affected by PE, as described above. Maternal socio-demographic characteristics and main laboratoristic findings are summarized in Table 2. All continuous variables showed a parametric distribution, except for maternal parity and maximum values of aspartate aminotransferase and 24 h proteinuria. As expected, patients with PE exhibited significantly higher values of both systolic and diastolic blood pressure, as well as 24 h proteinuria.

Neonatal outcomes in controls and cases are described in Table 3. Women affected by PE were more likely to deliver earlier, and their infants were significantly smaller than controls; a statistically significant difference was also observed in Apgar 5th.

### 3.2. Effect of Pravastatin on EGFL7 Expression in Ex Vivo Chorionic Villous Explant Cultures

**Figure 1:**
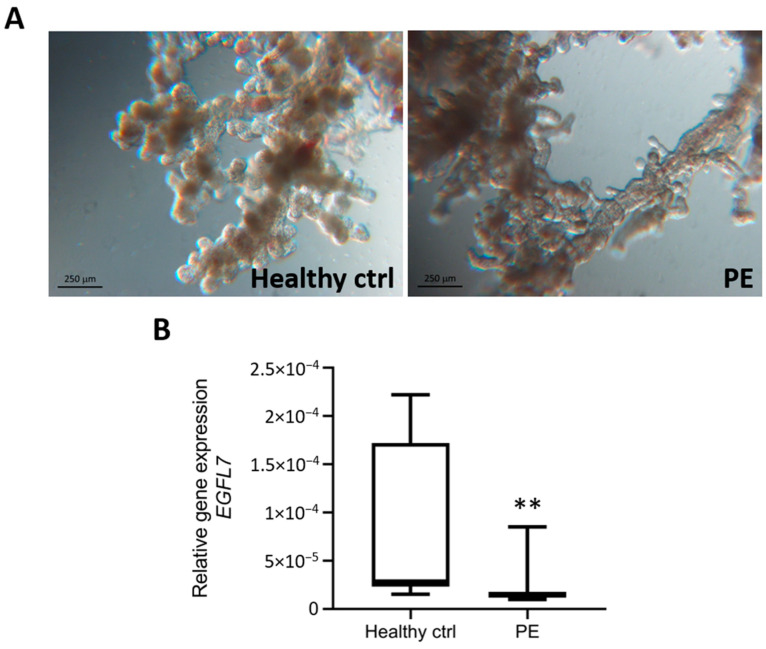
*EGFL7* gene expression levels in chorionic villous explants immediately after dissection. (**A**): Representative phase-contrast images of villous explant samples from control and PE placenta. (**B**): qRT-PCR analysis demonstrating reduced expression of *EGFL7* in PE villous explant samples compared to the healthy controls. Scale bar in panel images = 250 μM. Statistical analysis was performed using Mann–Whitney test (** *p* = 0.0044).

Placental levels of *EGFL7* were measured in chorionic villous explants from the control and PE placentas immediately after dissection. In agreement with what was previously reported [11], the qRT-PCR results demonstrated that *EGFL7* expression was significantly reduced in chorionic villi obtained from women with PE compared to the controls (*p* = 0.0044; Figure 1).

**Figure 2:**
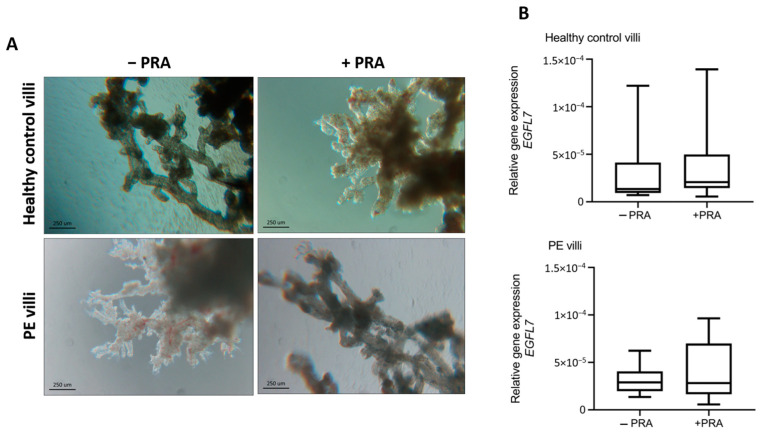
Effect of pravastatin administration to ex vivo chorionic villous explant cultures on *EGFL7* expression. (**A**): Representative phase-contrast images of villous explants from healthy control and PE placenta after 24 h of culture in the presence or absence of 10 μM pravastatin (PRA). (**B**): qRT-PCR analysis showing that PRA treatment did not affect *EGFL7* expression in both healthy control and PE villous cultures. Scale bar in panel images = 250 μM. Statistical analysis was performed using Student’s *t* test. − and + PRA: without or with 10 μM pravastatin.

Since PRA was recently evaluated for treatment and/or prophylaxis of PE, we tested whether PRA was able to modulate *EGFL7* expression. To this end, chorionic villous explants were cultured for 24 h with or without 10 μM PRA (Figure 2). qRT-PCR demonstrated that PRA did not affect its expression in healthy control villi, while it slightly increased *EGFL7* gene expression in villous cultures obtained from PE patients, although statistical significance was not met when PE patients were considered as one group (Figure 2B).

### 3.3. Different EGFL7 Expression Modulation after Pravastatin Treatment in Pre-Eclampsia Chorionic Villous Explant

**Figure 3:**
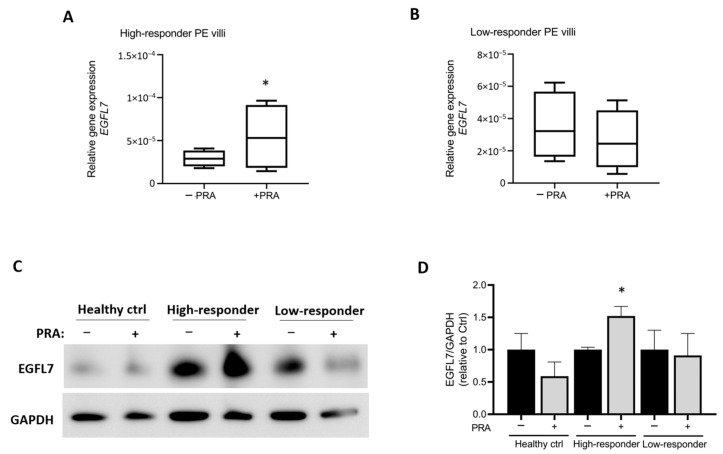
Identification of high-responder and low-responder PE pregnancies following treatment of chorionic villous explant cultures with 10 μM pravastatin (PRA) for 24 h. (**A**,**B**): qRT-PCR analysis demonstrating increased expression of *EGFL7* in high-responder PE villous explant cultures following PRA treatment (**A**) and no changes in low-responder PE villous cultures (**B**) when compared to villi cultured without PRA. (**C**): Western blot analysis of villous explant cultures with or without PRA, indicating increased expression of EGFL7 in high-responder PE villous cultures. (**D**): Quantification of Western blot analysis (*n* = 3). Statistical analysis was performed using Student’s *t* test (* *p* = 0.0467 for qRT-PCR; * *p* = 0.028 for western blot). − and + PRA: without or with 10 μM pravastatin.

According to the levels of *EGFL7* expression in villous cultures following PRA treatment, two groups of patients with pregnancies complicated by PE could be identified, which were classified into high and low responders. Chorionic villous cultures from the high-responders group exhibited significantly increased *EGFL7* gene expression after PRA treatment (n = 4, *p* = 0.0467; Figure 3A), whereas those from women identified as low responders showed no significant changes in *EGFL7* levels following PRA treatment (n = 4; Figure 3B). The significant increase in EGFL7 expression in villous explant cultures from high-responder PE patients was also confirmed at the protein level by Western blot analysis (*p* = 0.028, Figure 3C,D).

Based on these results, we investigated the maternal and biochemical characteristics of the two groups of PE patients (Table 4), as well as their perinatal outcomes and histopathological placental findings (Table 5 and Table 6). No statistically significant differences were noted between the two groups in terms of maternal characteristics or perinatal outcomes. In the low-responder group, all women presented with an abnormal second-trimester uterine artery mean pulsatility index, whereas in the high-responder group, this parameter was abnormal in only half of the cases. A significant difference was also detected in the pharmacological treatment performed in utero: 75% of the low-responder patients underwent prophylactic treatment with low-dose aspirin (LDA 150 mg/day) (*p* = 0.028), and 50% underwent prophylaxis with low-molecular-weight heparin (LMWH), whereas none of the high-responders received either aspirin or heparin during pregnancy. Among the low-responder patients, one had taken LDA alone for high risk of PE at the combined test; the other two were treated with LDA in association with LMWH for different clinical findings: the first one was diagnosed with antiphospholipid syndrome with persistently positive lupus anticoagulant and a previous history of both adverse obstetric and thrombotic events (intrauterine death and cerebral sinovenous thrombosis). The second one was affected by chronic hypertension and thrombophilia. In all these cases, LDA was correctly started before 14 weeks’ gestation.

Histopathological analyses of placental tissues showed villous hypoplasia in 75% of the samples in the low-responder group, a condition not observed in the placentas of high responders. Similarly, sites of placental infarcts were observed in 50% of the low-responder patients, but not in the high-responders group (Table 6).

### 3.4. Effect of Pravastatin Treatment on NOTCH1 Signaling Pathway

**Figure 4:**
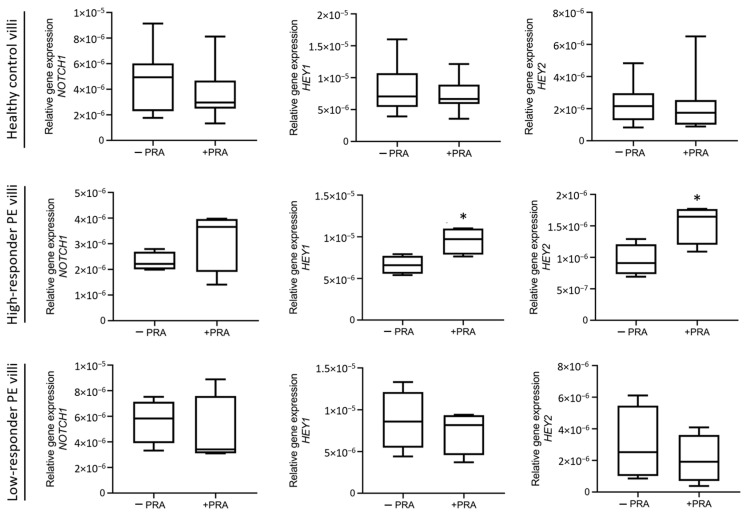
Expression analysis of *NOTCH1* and NOTCH target genes in chorionic villous explant culture samples from healthy control and high- and low-responder PE patients untreated or after pravastatin (PRA) treatment. qRT-PCR analysis of *NOTCH1*, hairy, and enhancer of split-related protein 1 (*HEY1*) and hairy and enhancer of split-related protein 2 (*HEY2*) gene expression in untreated or PRA-treated villi samples, indicating that PRA treatment significantly increased the expression of *NOTCH1* target genes in high-responder PE villous cultures, while it did not affect their expression in both healthy controls and low-responder PE villous cultures. Statistical analysis was performed using Student’s *t* test (* *p* = 0.0290 *HEY1*; * *p* = 0.0276 *HEY2*).

Activation of the NOTCH signaling pathway has been recognized as important for proper placental development and function [12,32,33]. In fact, expression of different Notch receptors, ligands, and targets is reduced in the placenta of women affected by PE [11,34,35]. Based on previous studies demonstrating that EGFL7 modulates NOTCH signaling [12], we investigated whether PRA treatment also affected this pathway. To this end, we performed qRT-PCR analyses for *NOTCH1* and its target genes hairy and enhancer of split-related protein 1 (*HEY1*) and hairy and enhancer of split-related protein 2 (*HEY2*) on chorionic villous explant cultures obtained from healthy controls and high and low-responder PE patients following PRA treatment. Our results show that *NOTCH1* and *HEY1* and *HEY2* were upregulated by PRA in high-responder PE (Figure 4); here, statistical significance was met for *HEY1* and *HEY2*, but not for *NOTCH1* (*p* = 0.0290, *p* = 0.0276 and *p* = 0.2166, respectively). Together, these data indicate that in the high-responder PE placentas, PRA treatment upregulates EGFL7 and activates the Notch pathway. In contrast, in villi obtained from healthy controls and low-responder PE placentas, PRA treatment did not affect *NOTCH1*, *HEY1*, and *HEY2* gene expression (Figure 4).

## 4. Discussion

In agreement with our previous study on whole placental tissue [11], here we show that expression of EGFL7 is strongly reduced in villi obtained from placentas of women affected by PE compared to healthy controls. When incubated with PRA, only a slight increase in *EGFL7* gene expression in villous cultures obtained from PE patients was observed. However, amongst the patients affected by PE, two different groups could be identified: a high-responder group, where chorionic villi exhibit significantly increased expression of EGFL7 after PRA incubation, and a low-responder group, which showed no significant changes in EGFL7 expression after PRA incubation.

For the first time, in the present study, we investigated the effects of PRA specifically on the expression of EGFL7. Indeed, EGFL7 should be considered part of the panel of pro- and anti-angiogenic factors, which are known to be dysregulated in IUGR and PE [36]. In fact, while EGFL7 is highly downregulated in placental tissues of women with PE [11], circulating levels of EGFL7 are more than three times higher in women affected by early-onset PE compared to controls and isolated IUGR. Moreover, the dosage of circulating levels of EGFL7 has an additional diagnostic and prognostic value in comparison to s-Flt1 and PlGF or s-Endoglin (sEng), since it is not only detectable in maternal blood before the clinical manifestation of PE, but it also allows us to efficiently discriminate between pregnancies affected by PE and those with isolated IUGR [13,37]. We hypothesized that the increased circulating levels of EGFL7 are the direct result of maternal systemic involvement and diffuse endothelial damage specific to PE, which is not present in controls and isolated IUGR. These conditions, i.e., healthy pregnancy and isolated IUGR, are characterized by low detectable maternal circulating levels of EGFL7 throughout gestation [36,37].

For all these reasons, we tested the effect of PRA on EGFL7 expression. The ability of PRA to ameliorate or prevent the clinical scenario of PE by modulating the expression of angiogenetic factors has emerged in several in vitro and in vivo studies [38,39,40,41]. In vitro studies have demonstrated the ability of PRA to reduce sFlt1 secretion by human umbilical venous endothelial cells, trophoblast cells, and placental explants taken from women affected by PE; moreover, PRA has been shown to reduce oxidative stress by activating the heme-oxygenase 1 enzyme [40,41]. Studies on mouse models of PE have shown that PRA is able to reduce the secretion of sFlt1 and increase the secretion of PlGF, improving the clinical phenotype of the disease [38]. It has been shown that treatment with PRA results in increased proliferation, migration, and tube formation ability of umbilical-cord-blood-derived endothelial colony-forming cells (ECFCs), which are a highly proliferative subtype of endothelial progenitor cells (EPCs). These effects were accompanied by augmented AKT- and eNOS-phosphorylation, increased expression levels of heme oxygenase-1 (HO-1), vascular endothelial growth factor A (VEGF-A), and PlGF and decreased expression levels of sFlt-1 and Eng [15]. These studies have contributed to a better understanding of the pleiotropic function of statins and have also provided a promising basis for the use and role of PRA in the treatment and prevention of PE [20,42].

Our study initially investigated the ability of PRA to induce EGFL7 expression by placental explants taken from women affected by PE, considering all patients as one group. Interestingly, not all chorionic villi showed a comparable response to PRA. Indeed, we identified one group, the low-responder group, which, despite having comparable maternal and perinatal conditions, did not show increased expression of EGFL7 following PRA treatment. It is noteworthy that this group mostly comprises women who, despite correctly following the pharmacological prophylaxis with LDA (75% of cases) and LMWH (50% of cases) due to high-risk PE medical conditions, still developed the early and more severe form of the disease, complicated by IUGR, maternal and fetal placental malperfusion, and preterm delivery [43,44,45]. Another group of patients was identified, whom we called high responders, for which PRA was able not only to induce the expression of EGFL7, but also to activate the Notch signaling pathway, as demonstrated by the increased expression of the Notch target genes *HEY1* and *HEY2*. The concomitant upregulation of EGFL7 and activation of the Notch pathway has been previously shown in trophoblast cells and in other cell systems [12]. Our studies suggest a potential novel molecular mechanism underlying the effect of PRA on chorionic villi, a hypothesis that is further supported by previous observations that statins are able to regulate Notch signaling activity [46,47,48].

Our data are the first to reveal that in the group where standardized pharmacological prophylaxis has failed in preventing PE, PRA was also unable to modulate EGFL7 expression. It is possible that the failure to induce EGFL7 expression may be a consequence of the treatments administered during pregnancy. However, this hypothesis does not apply to all patients as not all were treated. Alternatively, our findings may reflect an aspect of the still unknown and unexplained reasons some patients do not respond to conventional pharmacological prophylaxis. PRA belongs to the pharmacological class of statins which, in addition to their lipid-lowering properties, may also exhibit some anti-inflammatory effects [49], including reduction in the adhesion of inflammatory cells by diminishing the expression of nuclear factor-kappa B (NF-kB), activator protein-1, and vascular cell adhesion molecule (VCAM), which are molecular pathways also regulated by EGFL7, in order to reduce endothelial cell activation in normal and inflammatory conditions [50,51,52,53,54]. This shared mechanism of action may be one possible explanation for our results. Clinically, we observed that LDA, sometimes associated with LMWH, did not prevent the onset of PE. Additionally, in vitro, in the same women, PRA failed to restore the expression of EGFL7 in chorionic villi, which is crucial for maintaining proper endothelial and trophoblast functions.

The main limitation of our study is the small number of placental villi analyzed that prevented us from making final conclusions. This was, in part, due to the experimental procedure, that requires setting up chorionic villous explant cultures soon after delivery, which was often occurring in conditions of emergency for PE patients. The small number of samples was also due to the strict inclusion criteria, which, on the other hand, represents the main strength of this study. The restricted inclusion/exclusion criteria allowed us to reduce the incredibly high number of clinical and biological variables related to pregnancy, which otherwise would have further complicated the interpretations of the results.

## 5. Conclusions

In conclusion, our study identified the modulation of EGFL7 levels as a potential new mechanism of action for PRA, suggesting a novel potential pharmacological approach for the prevention and treatment of PE.

We demonstrated that PRA treatment of placental villi effectively regulates EGFL7 expression, which is normally significantly reduced in PE placentas and elevated in the blood of PE patients compared to controls. Restoring EGFL7 to its physiological levels could thus provide a solid biological basis for using this new pharmacological strategy against PE.

Additionally, our research identified a subgroup of PE patients, termed “low responders”, in which prophylaxis with LDA, sometimes combined with LMWH, failed to prevent PE. In these patients, PRA also failed to modulate EGFL7 expression in chorionic villous cultures, suggesting a likely failure in preventing PE as well. A prospective study is warranted to identify these high-risk women early, given the ineffectiveness of the current pharmacological approaches, and to explore new potential prevention strategies.

Our research results not only support the use of PRA in the prevention of PE, as suggested by some clinical trials, but also highlight the importance of early and, if possible, pre-conceptional assessment of high-risk women. This assessment aims to identify candidates who may not respond to standard pharmacological prophylaxis. By focusing on these individuals, we can better understand the molecular and clinical mechanisms underlying the failure of conventional treatments and develop personalized and tailored approaches.

## Figures and Tables

**Table 1:** Primer sequences, amplicon size, and gene accession number.

Gene	Primer Sequence	AL (bp)	Accession No.
*EGFL7* (forward)	5′-TCGTGCAGCGTGTGTACCAG-3′	92	NM_016215.5
*EGFL7* (reverse)	5′-GCGGTAGGCGGTCCTATAGATG-3′		
*NOTCH1* (forward)	5′- GCGGGATCCACTGTGAGAA -3′	58	NM_0176617.5
*NOTCH1* (reverse)	5′- CCGTTGAAGCAGGAGCTCTCT -3′		
*HEY1* (forward)	5′-CATCGAGGTGGAGAAGGAGAGT-3′	66	NM_012258.4
*HEY1* (reverse)	5′-GACATGGAACCTAGAGCCGAACT-3′		
*HEY2* (forward)	5′-CGACCTCCGAGAGCGACAT-3′	67	NM_012259.3
*HEY2* (reverse)	5′-CTTTGCCCCGAGTAATTGTTCT-3′		
*18S* (forward)	5′-GAGGCCCTGTAATTGGAATGAG-3′	120	NR_145820
*18S* (reverse)	5′-GCAGCAACTTTAATATACGCTATTGG-3′		

AL (bp): amplicon length (base pair).

**Table 2:** Maternal socio-demographic characteristics and laboratory findings of controls (*n* = 10) and cases (*n* = 8).

Variable	Controls (*n* = 10)	PE Patients (*n* = 8)	*p*-Value
Maternal age (years)	34.60 ± 1.13	31.50 ± 2.51	0.244
Parity	0.00 (0.00)	0.00 (0.25)	0.408
Pre-conceptional BMI	21.64 ± 8.43	24.35 ± 14.63	0.131
Gestational weight gain	11.62 ± 1.45	12.00 ± 1.00	0.835
Second Trimester mean uterine arteries PI > 95° pc	3/10 (30.00%)	5/8 (62.50%)	0.342
Maximum SBP (mmHg)	115.50 ± 2.83	164.63 ± 6.92	**<0.0001**
Maximum DBP (mmHg)	71.20 ± 2.31	101.63 ± 4.73	**<0.0001**
Minimum PLT count	219.400 ± 14.740	190.630 ± 24.530	0.309
Maximum Creatinine (mg/dL) level	0.43 ± 0.08	0.66 ± 0.09	0.092
Maximum AST (UI/L) level	16.00 (18.00)	14.00 (28.50)	0.965
Maximum LDH (mg/dL) level	214.75 ± 17.68	245.50 ± 19.98	0.268
Maximum 24 h proteinuria (gr/L) level	0.00 (0.00)	1.50 (7.20)	**0.006**

Comparison between controls and cases; PE: pre-eclampsia; BMI: body mass index; PI: pulsatility index; SBP: systolic blood pressure; DBP: diastolic blood pressure; PLT: platelets; AST: aspartate aminotransferase; LDH: lactate dehydrogenase; The bold indicates significant values.

**Table 3:** Perinatal and neonatal outcome (The bold indicates significant values).

Variable	Controls (*n* = 10)	PE Patients (*n* = 8)	*p*-Value
Gestational age at delivery (weeks)	38.78 ± 16.54	31.66 ± 8.74	**<0.0001**
Birthweight (g)	3547.00 ± 138.13	1277.50 ± 117.59	**<0.0001**
Birthweight centile	66.70 ± 9.18	3.57 ± 1.69	**<0.0001**
Apgar 5th	9.56 ± 0.18	8.63 ± 0.18	**0.002**

**Table 4:** Maternal socio-demographic characteristics and biochemical findings of women with pre-eclampsia according to EGFL7 expression in response to pravastatin treatment.

Variable	Low-Responder (*n* = 4)	High-Responder (*n* = 4)	*p*-Value
Maternal age (years)	35.50 ± 2.22	27.50 ± 3.71	0.114
Parity	0.50 (1.00)	0.00 (0.00)	0.343
Pre-conceptional BMI	25.69 ± 2.42	23.02 ± 1.71	0.402
Gestational weight gain	12.25 ± 1.60	11.75 ± 1.44	0.824
LDA	3/4 (75%)	0/4 (0%)	**0.028**
LMWH	2/4 (50%)	0/4 (0%)	0.102
Uterine arteries mean PI > 95° pc	4/4 (100%)	2/4 (50%)	n.a.
Maximum SBP (mmHg)	163.75 ± 6.54	165.50 ± 13.43	0.911
Maximum DBP (mmHg)	102.50 ± 7.26	100.75 ± 7.16	0.869
Minimum PLT count	184.250 ± 40.171	197.000 ± 68.327	0.817
Creatinine (mg/dL) maximum level	0.59 ± 0.18	0.72 ± 0.99	0.589
Maximum AST (UI/L) level	47.50 (108.75)	11.00 (3.00)	0.200
LDH (mg/dL) maximum level	236.00 ± 17.34	255.00 ± 38.76	0.670
Proteinuria maximum (gr/L)	2.95 (7.20)	1.50 (4.93)	0.686

BMI: body mass index; LDA: low-dose aspirin; LMWH: low-molecular-weight heparin; SBP: Systolic blood pressure; DBP: Diastolic blood pressure; PLT: platelets; AST: aspartate aminotransferase; LDH: lactate dehydrogenase; The bold indicates significant values.

**Table 5:** Perinatal and neonatal outcome of women with pre-eclampsia according to EGFL7 expression in response to pravastatin treatment.

Variable	Low-Responder (*n* = 4)	High-Responder (*n* = 4)	*p*-Value
Gestational age at delivery (weeks)	32.00 ± 1.67	31.32 ± 0.84	0.728
Birthweight (g)	1305.00 ± 233.00	1250.00 ± 98.68	0.835
Birthweight centile	1.65 ± 0.62	5.45 ± 3.25	0.289
Apgar 5th	8.50 ± 0.29	8.75 ± 0.25	0.537

**Table 6:** Histopathological findings of placental tissues obtained of women with pre-eclampsia.

Maternal Vascular Perfusion	Low-Responder (*n* = 4)	High-Responder (*n* = 4)
Infarcts	2/4 (50%)	0/4 (0%)
Accelerated villous maturation	4/4 (100%)	4/4 (100%)
Villar agglutination	2/4 (50%)	3/4 (75%)
Villar hypoplasia	3/4 (75%)	0/4 (0%)
Increased syncytial knots	4/4 (100%)	4/4 (100%)
**Fetal Vascular Perfusion**	**Low-Responder (*n* = 4)**	**High-Responder (*n* = 4)**
Avascular villi	3/4 (75%)	1/4 (25%)
Intramural fibrin deposition	1/4 (25%)	0/4 (0%)
Stem vessel obliteration	0/0 (0%)	0/0 (0%)
Thrombosis	0/0 (0%)	0/0 (0%)

Placental histopathology analyzed according to the Amsterdam Placental Workshop Group Consensus Statement [26].

## Data Availability

The data presented in this study are available on request from the corresponding author.
